# On the Role of Iron in Idiopathic Parkinson’s Disease

**DOI:** 10.3390/biomedicines11113094

**Published:** 2023-11-20

**Authors:** Sandro Huenchuguala, Juan Segura-Aguilar

**Affiliations:** 1 Escuela de Tecnología Médica, Facultad de Salud, Universidad Santo Tomás, Santiago 8370003, Chile; sandrodemian@gmail.com; 2 Molecular & Clinical Pharmacology, Institute of Biomedical Sciences (ICBM), Faculty of Medicine, University of Chile, Santiago 8380453, Chile

**Keywords:** iron, chelator, deferiprone, dopamine, neuromelanin, neurodegeneration, neuroprotection, oxidative stress, MPTP, Parkinson’s disease

## Abstract

The transition metal characteristics of iron allow it to play a fundamental role in several essential aspects of human life such as the transport of oxygen through hemoglobin or the transport of electrons in the mitochondrial respiratory chain coupled to the synthesis of ATP. However, an excess or deficiency of iron is related to certain pathologies. The maintenance of iron homeostasis is essential to avoid certain pathologies related to iron excess or deficiency. The existence of iron deposits in postmortem tissues of Parkinson’s patients has been interpreted as evidence that iron plays a fundamental role in the degenerative process of the nigrostriatal system in this disease. The use of iron chelators has been successful in the treatment of diseases such as transfusion-dependent thalassemia and pantothenate kinase-associated neurodegeneration. However, a clinical study with the iron chelator deferiprone in patients with Parkinson’s disease has not shown positive effects but rather worsened clinical symptoms. This suggests that iron may not play a role in the degenerative process of Parkinson’s disease.

## 1. Iron

**Figure 1:**
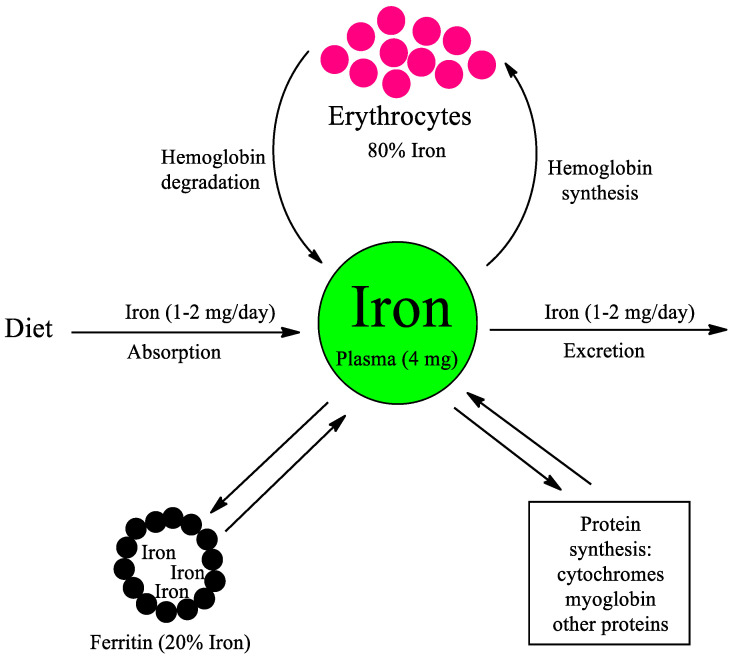
Iron homeostasis. Iron has a fundamental role in the human body to avoid different pathologies. Most iron (80%) is found in erythrocytes where hemoglobin is located. Hemoglobin has a half-life of 120 days, which implies that there is a permanent degradation and synthesis of hemoglobin. Iron homeostasis is also regulated through dietary iron intake and excretion. An amount of 20% of iron is accumulated in the protein ferritin. Iron, to a lesser extent, is used for the synthesis of proteins such as cytochromes, myoglobin, and other proteins.

Iron is a transition metal that is essential for human life due to its ability to change redox states where reduced iron (Fe^2+^) can be oxidized to oxidized iron (Fe^3+^). An iron molecule is in the center of the protoporphyrin IX molecule, which is the catalytic component of the heme group. The heme group exists in different proteins where iron can change its redox state. In hemoglobin, iron plays an essential role for the life of the human being since it can bind dioxygen, transporting it through the bloodstream throughout the body and then releasing it in the tissues that require it. The heme group also plays a catalytic role in the cytochrome P450 superfamily in the synthesis of cholesterol, vitamin D, and hormones such as testosterone and estrogens. Another important role of cytochrome P450 is the detoxification of xenobiotics, which includes drugs, which increases with age as diseases increase. About 70–80% of the iron in humans is found in proteins that have a heme group such as hemoglobin, myoglobin, and cytochromes. The rest is stored in the protein ferritin, which is where iron is stored, around 20% of the total iron. Apoprotein ferritin is composed of 24 subunits of 18,500 Dalton that surround, in micellar form, around 3000 to 4000 iron atoms in its oxidized state (Fe^3+^) and has a molecular weight of 440,000 Dalton (Figure 1).

Biological reducing agents such as ascorbic acid, reduced flavin, and N-acetylcysteine can release the oxidized iron fixed in ferritin in its reduced form (Fe^2+^). Iron in its reduced state catalyzes the Fenton reaction, in which hydrogen peroxide is converted to a hydroxide ion and a hydroxyl radical, which is a potent radical that induces oxidative stress [1,2,3,4].

The major source of iron in the human body is obtained from the recycling of iron from hemoglobin where macrophages break down senescent red blood cells. Another source of iron is dietary iron where divalent metal transporter 1 expressed in enterocytes takes up dietary iron and is then transported by ferroportin into the blood. This circulating reduced iron is taken up by ferritin after its oxidation to Fe3+ catalyzed by ceruloplasmin. Circulating transferrin is taken up by bone marrow cells, hepatocytes, and macrophages that express transferrin receptor 1 on their plasma membrane. Most of the iron is in hemoglobin while ferritin is the major source of iron found in the liver. There is a fine regulation of iron levels where an increase in iron in the hepatocytes triggers the secretion of hepcidin, which induces the internalization and degradation of ferroportin to decrease and control the transport of iron from the enterocytes to the blood circulation [2,5].

There is post-transcriptional regulation at the cellular level of ferritin and transferrin receptors based on the mRNA stability of these proteins. This regulation is mediated by the existence of iron regulatory proteins (IRPs) that change their configuration according to the levels of the intracellular iron pool. IRPs have a high affinity with an mRNA sequence of these proteins called an iron responsive element (IRE) that is in the untranslated region. When intracellular iron pool levels are high, iron induces a change in the IRP configuration that prevents its binding with an IRE, which, in ferritin mRNA, is located before the sequence that encodes the expression of this protein, resulting in an increased expression of ferritin. On the other hand, under low levels of intracellular iron, an IRP binds to a transferrin IRE, inhibiting its expression [2,6].

Iron homeostasis in the human body is essential for its health since both iron deficiency and excess are implicated in different pathologies. Anemia is a disease caused by iron deficiency that especially affects women with excessive menstruation. It also affects children and populations with low and medium income. Hemochromatosis is an autosomal recessive hereditary disease caused by a mutation in the hemochromatosis protein gene that induces an increase in the absorption of dietary iron. Excess iron is not bound to the protein transferrin and is taken up by hepatocytes, cardiomyocytes, and pancreatic islet cells. This disease affects different organs such as the pancreas, heart, liver, skin, thyroid, pituitary, joints, and gonads. Excess iron also generates liver fibrosis because of the presence of excess iron deposits in the extracellular matrix, which is a consequence of hereditary hemochromatosis [7,8,9,10,11].

**Figure 2:**
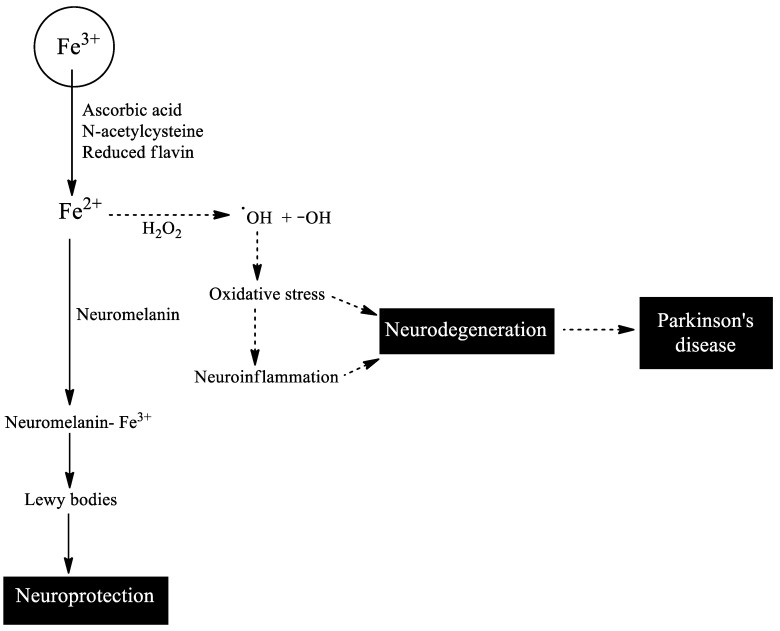
The possible fate of reduced iron in neuromelanin-containing dopaminergic neurons. Iron bound to proteins is in its oxidized state (Fe^3+^) but reducing agents such as ascorbic acid, reduced flavin, or N-acetylcysteine can release it in its reduced state (Fe^2+^). Fe^2+^ catalyzes the formation of hydroxyl radicals in the presence of hydrogen peroxide, which is generated in the oxidative deamination of dopamine to 3,4-dihydroxyphenylacetaldehyde catalyzed by monoamine oxidase in dopaminergic neurons. This oxidative stress can induce neurodegeneration of neuromelanin-containing dopaminergic neurons and/or induce neuroinflammation that ultimately also induces neurodegeneration. This degenerative process results in the loss of neuromelanin-containing dopaminergic neurons in Parkinson’s disease. However, neuromelanin-containing dopaminergic neurons produce neuromelanin that functions as an iron chelator, preventing the neurotoxic effects of Fe^2+^ in the presence of hydrogen peroxide. Finally, this neuromelanin containing Fe^3+^ is deposited in Lewy bodies.

Neuromelanin also contributes to the maintenance of a balance in iron levels in neuromelanin-containing dopaminergic neurons thanks to its ability to function as an endogenous chelator [12,13,14,15]. Neuromelanin is a pigment that is formed in dopaminergic neurons during the oxidation of the catechol group of dopamine to neuromelanin, and its concentration depends on the level of expression of the vesicular monoamine transporter 2, which transports dopamine to the monoaminergic neurotransmission vesicles [16,17,18]. Dopamine is oxidized to three transient ortho-quinones in a sequential manner: dopamine → dopamine-ortho-quinone → aminochrome → 5,6-indolequinone → neuromelanin [19]. The polymerization of 5,6-indolequinone to neuromelanin generates a dark pigment that can be seen in the substantia nigra and is deposited in independent double-membrane structures [19]. Free neuromelanin activates microglia and induces the loss of dopaminergic neurons in the substantia nigra of treated animals [12,20,21]. However, the formation of neuromelanin is considered a normal and harmless process since dopaminergic neurons loaded with neuromelanin are intact in healthy older adults at the time of death [22,23]. Neuromelanin increases with age and plays an important role in chelating free iron that can induce neurotoxic effects to maintain iron homeostasis [24] (Figure 2).

## 2. Iron Toxicity

Excess iron in different tissues and its reduction generate free Fe^2+^ compared to that in the presence of hydrogen peroxide that catalyzes the formation of hydroxyl radicals [25,26,27]. Excess iron in the brain impairs hippocampal neurogenesis by decreasing the number of new neurons through affecting the iron-BDNF-furin pathway [28]. Iron induces the activation of BV2 microglia; the increase in reactive oxygen species; and the activation of cytokines, TNF-a, and IL-1B. This is accompanied by a decrease in the potential of mitochondrial membranes and an increase in the activity of proteins related to apoptosis such as Bcl2, BAx, P53, and caspase 9 [29]. Excess iron induces a loss of cognitive functions and loss of synaptic proteins that is attenuated by ferrostatin-1, a ferroptosis inhibitor, and z-VAD-FMK, a pan-caspase inhibitor [30]. The reduced release of iron from damaged erythrocytes in the hematoma is essential for brain damage in intracerebral hemorrhage [31].

Ferroptosis, an iron-induced programmed death, is involved in several pathologies. In traumatic brain injury, ferroptosis induces iron accumulation, the formation of reactive oxygen species, the disruption of iron metabolism, and changes in mitochondria morphology [32]. In hypoxic–ischemic brain damage, iron accumulates in the cerebral cortex because of the destruction of red blood cells that triggers ferroptosis, inducing shrunken mitochondria, increased lipid peroxidation, and reduced glutathione peroxidase 4 and solute carrier family 7 member 11 [33]. It has been proposed that the iron-dependent activation of redox-sensitive Ca^2+^ channels could be involved in ferroptosis [34]. It has been suggested that alpha-synuclein dysfunction could be related to ferroptosis due to the functional relationship of alpha-synuclein with iron metabolism and lipid peroxidation [35]. A study using human-induced pluripotent-stem-cell-derived microglia demonstrated that these cells are very sensitive to iron accumulation and induce ferroptosis. Iron accumulation in these cells induces a change in transcriptional status that matches the transcriptional status of microglia from postmortem material from brains of Parkinson’s disease patients [36]. In subarachnoid hemorrhage, the activation of M1 microglia plays an important role in neuroinflammation. The enzyme nitric oxide synthase promotes the survival of M1 microglia and inhibits ferroptosis. The nitric oxide synthase inhibitor decreases the number of M1 microglia, decreasing neuroinflammation under these conditions and promoting ferroptosis [37].

Lutein, which has been shown to have protective effects on oxidative stress and inflammation, attenuates glutamate-induced inflammation, iron accumulation, lipid peroxidation, and reactive oxygen species formation [38]. Human neuromelanin isolated from substantia nigra protects against iron-dependent hydroxyl radical production under iron-accumulating conditions [15]. Studies with Ginkgolide B have shown to have an impact on the levels of ferroptosis markers such as reactive oxygen species, superoxide dismutase, malondialdehyde, reduced iron, glutathione peroxidase 4, nuclear receptor coactivator 4, ferritin heavy polypeptide 1, and acyl-CoA synthetase long-chain family member 4 in cerebral I/R injury [39]. Berberine decreases the effects of ferroptosis by decreasing reactive oxygen species, lipid peroxidation, and iron levels while increasing levels of glutathione peroxidases 4, glutathione, superoxide dismutase, and cystine/glutamate antiporter SLC7A11 [40]. In experiments with cardiac deficiency, ferroptosis was inhibited when the Sirt1/p53 pathway was activated. Sirt1 increases the levels of glutathione peroxidase 4, reduces glutathione, and decreases the acetylation of p53 K382, decreasing the degradation of SLC7A11 [41]. Puerarin has been shown to inhibit ferroptosis in autism spectrum disorder through the inhibition of iron accumulation, lipid peroxidation, and mitochondrial impairment. Puerarin also increases the expression of proteins that inhibit ferroptosis such as glutathione peroxidase 4, nuclear factor erythroid 2-related factor 2, sodium-independent cystine-glutamate antiporter, and ferritin heavy chain 1 [42].

## 3. Iron and Parkinson’s Disease

A study carried out with 101 patients with Parkinson’s disease with quantitative susceptibility mapping data concluded that the ventral tegmental area does not show a significant increase in iron during pre-motor symptoms but that it significantly increases when the disease progresses [43]. Alpha lipoic acid, an iron chelating agent and antioxidant, decreases motor deficiencies in a Parkinson’s animal model, and increases ferritin heavy chain 1 and ferroportin expression while decreasing divalent metal transporter 1 expression. Alpha lipoic acid decreases lipid peroxidation, mitochondrial damage, and the accumulation of reactive oxygen species, and inhibits ferroptosis by increasing the expression of glutathione peroxidase 4 and the cysteine/glutamate transporter [44]. A study carried out in BV2 microglia demonstrated that both monomeric and oligomeric alpha-synuclein catalyze the reduction of Fe^3+^ to Fe^2+^ but that this activity is more increased in oligomeric alpha-synuclein. The two forms of alpha-synuclein are capable of activating microglia and pro-inflammatory factors and increasing the expression of ferroportin 1, divalent metal transporter 1 [45]. Iron accumulation activates microglia, generating a metabolic change from oxidative phosphorylation to glycolysis that polarizes them, adopting a proinflammatory M1 phenotype that induces neuroinflammation and neurodegeneration [46]. A meta-analysis determined the correlation between iron content and Parkinson’s disease by using the magnetic resonance imaging technique and markers of iron metabolism in cerebrospinal fluid and blood. Iron levels determined via magnetic resonance imaging are increased in the substantia nigra of patients with Parkinson’s disease. However, there is no difference between patients with Parkinson’s disease and control subjects in serum/plasma/cerebrospinal fluid iron, ferritin, and transferrin levels [47].

For many years, the existence of iron deposits in the brain of a Parkinson’s patient has been considered [48,49] as evidence of the role of iron in the degenerative process of the nigrostriatal dopaminergic system, which ends with the loss of neuromelanin-containing dopaminergic neurons. When there is a degenerative process that ends with the death of a neuron, what takes place is that the microglia appear and phagocytize all the remains of this neuron in the process of death. Therefore, all the proteins, organelles, and membranes of this neuron that has died are removed and, after years of the degenerative process in a patient with PD, the postmortem tissue only shows the neuronal and glial cells that have survived this degenerative process during years [50]. Therefore, the existence of iron deposits in the postmortem material does not imply that iron has triggered a degenerative process of neuromelanin-containing dopaminergic neurons. The question is the role of iron accumulation in the substantia nigra in patients with this disease. If iron accumulation in postmortem material from patients with Parkinson’s disease is not evidence for neurodegeneration, this iron accumulation possibly has a neuroprotective role because (i) we have never seen a patient with Parkinson’s disease exposed to high concentrations of iron, and (ii) in Huasco, a port city and commune in the Norte Chico of Chile with 9000 inhabitants, it received trains loaded with iron ore from the mines located in the mountains several times a day for decades. The iron ore came in the open wagons of the train that, as it passed, sent out powdered iron ore that all the inhabitants of this city inhaled several times a day for decades. However, despite this daily iron ore exposure for decades, there was no explosive increase in Parkinson’s disease in Huasco, suggesting that iron does not induce Parkinsonism.

## 4. Clinical Study with the Iron Chelator Deferiprone

The iron chelator deferiprone is successfully used in the treatment of transfusion-dependent thalassemia to avoid the toxic effects of iron overload [51]. The rare genetic disease pantothenate kinase-associated neurodegeneration is associated with iron accumulation in the brain and dystonia. A clinical study with these patients treated with deferiprone demonstrated a decrease in iron levels in the basal ganglia and slows the progression of this disease [52]. It has been proposed that the interaction of iron with alpha-synuclein and dopamine and its action on dopamine metabolism could play an important role in the degenerative process of the nigrostriatal system in Parkinson’s disease [53]. The therapeutic use of iron chelators in the treatment of Parkinson’s disease has been proposed based on the accumulation of iron and the oxidative stress observed in the disease. Using the preclinical model with animals treated with 1-methyl-4-phenyl-1,2,3,6-tetrahydropyridine (MPTP), it was observed that the iron chelator deferiprone induces the appearance of new tyrosine hydroxylase, increases the length of the axodendritic tree, and has a neuroprotective effect against lipid peroxidation [54] (Table 1).

Based on the increase in iron in the substantia nigra of patients with Parkinson’s disease, a phase 2 clinical study was conducted with 372 participants who received 15 mg/kg deferiprone or a placebo dose. Parkinson’s disease patients never received levodopa treatment, did not demonstrate positive effects with deferiprone treatment, and instead worsened compared with control subjects [55]. The failure of this clinical study with deferiprone can possibly be explained because it is based on two wrong assumptions: (i) Considering that the accumulation of iron in the substantia nigra is an indicator of neurodegeneration of neuromelanin-containing dopaminergic neurons, for many years, the discovery of iron deposits in postmortem tissues of patients with Parkison’s disease has been considered as evidence of the role of iron in the degenerative process of the disease. However, in our opinion, the existence of iron deposits in the surviving tissue demonstrates the opposite because neuromelanin-containing dopaminergic neurons that degenerated for years were phagocytosed by microglia. (ii) The preclinical studies that supported the conduct of clinical studies with patients with Parkinson’s disease do not present what occurs in Parkinson’s disease. The preclinical studies based on 1-methyl- 4-phenyl-1, 2, 3, 6-tetrahydropyridine (MPTP) demonstrated an extremely fast, massive, and propagative death in animals, which is completely the opposite of what occurs in the disease. In addition, MPTP in humans who consumed synthetic drugs contaminated with MPTP develop severe Parkinsonism in only three days [56]. There is a long list of failed clinical studies that were based on exogenous neurotoxins such as MPTP, which do not represent what occurs in the disease degenerative process [57,58,59,60,61].

**Table 1:** Clinical studies with deferiprone in different pathologies. The USA Food and Drug Administration approved in 2011 the use of the iron chelator deferiprone in the treatment of transfusional iron overload in thalassemia syndromes, sickle cell disease, or other anemias. In pathologies dependent on iron accumulation, the chelator deferiprone has a positive effect, while in Parkinson’s disease, in which the role of iron in the progression of the disease is unclear, deferiprone has a negative effect.

Disease	Aim of Treatment	Result	Reference
Transfusion-dependent thalassemia	Prevent the toxic effects of iron overload	Positive	Elalfy et al., 2023 [51]
Pantothenate kinase-associated neurodegeneration	Prevent iron accumulation	Positive	Klopstock et al., 2019 [52]
Transfusional iron overload in sickle cell disease	Prevent iron accumulation	Positive	Kwiatkowski et al., 2022 [62]
Friedreich’s ataxia	Prevent iron accumulation	Positive	Pandolfo M, Hausmann, 2013 [63]
Transfusion-dependent haemoglobinopathies-2	Prevent iron accumulation	Positive	Maggio et al., 2020 [64]
Parkinson’s disease	Prevent iron-dependent disease progression	Negative	Devos et al., 2022 [55]

## 5. The Degenerative Process in Parkinson’s Disease

The extremely rapid and massive action of MPTP in these individuals contrasts with the extremely slow rate at which the disease progresses. It takes years for 60% of neuromelanin-containing dopaminergic neurons to degenerate and motor symptoms to develop, while in patients intoxicated with MPTP, almost 90% of neuromelanin-containing dopaminergic neurons degenerate in just three days. Recently, it was estimated that the number of NM dopaminergic neurons that exist in the substantia nigra pars compacta is between 800,000 and 1,000,000, counting the two hemispheres of the human brain [65]. At the time motor symptoms appear, a patient should have between 320,000 and 400,000 surviving NM dopaminergic neurons, and if a patient with idiopathic Parkinson’s survives 15 years, then 58 to 73 NM dopaminergic neurons are lost every day. In the event that the patient with idiopathic Parkinson’s survives 10 years after the onset of motor symptoms, this implies that they lose between 88 and 110 neuromelanin-containing dopaminergic neurons per day. This slow rate of degeneration in idiopathic Parkinson’s disease suggests that this degenerative process may not be an expansive, massive, and rapid as that induced by MPTP. Single-neuron neurodegeneration has been proposed as a degenerative model of idiopathic Parkinson’s disease based on the extremely slow degenerative process observed in idiopathic Parkinson’s disease that takes years [23,50].

**Figure 3:**
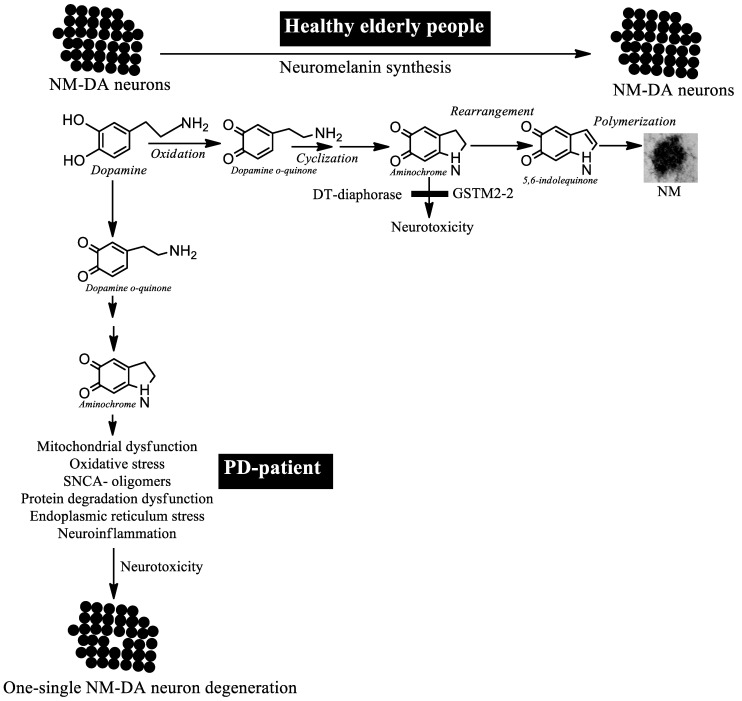
One-single neuromelanin-containing dopaminergic neuron degeneration model. The formation of neuromelanin is a normal and harmless process, although it requires the formation of three ortho-quinones that are potentially neurotoxic, of which aminochrome is the most neurotoxic. Healthy elderly people synthesize neuromelanin without the neurotoxic effects of aminochrome because there are enzymes DT-diaphorases and glutathione transferase M2-2 (GSTM2) that prevent the neurotoxic effects of aminochrome. In patients with idiopathic Parkinson’s disease, aminochrome levels exceed the neuroprotective capacity of DT-diaphorase and GSTM2, inducing the degeneration of a single neuromelanin (NM)-containing dopaminergic neuron.

The single-neuron degenerative model is based on the fact that the degenerative process is triggered by an endogenous neurotoxin that is formed in dopaminergic neurons that contain neuromelanin and that it does not have an expansive effect. This degenerative process induces the death of a single neuron without affecting neighboring neurons. In this single-neuron degeneration, the accumulation of dopaminergic neurons that are lost is very slow, which explains the failure of preclinical models with neurotoxins such as 6-hydroxydopamine and MPTP, since they do not measure a therapeutic effect in such a slow degenerative process (Figure 3).

**Figure 4:**
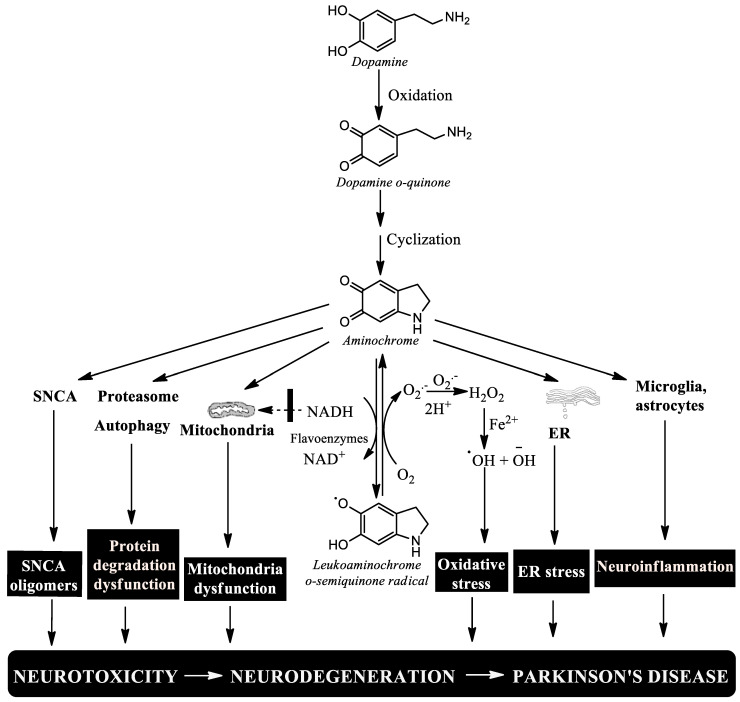
Aminochrome-induced neurotoxicity. Aminochrome is the most stable and neurotoxic ortho-quinone formed during neuromelanin synthesis. Aminochrome is neurotoxic by inducing the formation of neurotoxic alpha-synuclein oligomers, the protein degradation dysfunction of both the proteasomal and lysosomal systems, mitochondrial dysfunction, oxidative stress, endoplasmic reticulum stress, and neuroinflammation.

Possible endogenous neurotoxins that are generated in dopaminergic neurons and that could induce single-neuron degeneration include alpha-synuclein, 3,4-dihydroxyphenylacetaldehyde (DOPAL), free neuromelanin, and aminochrome [66]. Alpha-synuclein can be added to fibrils or oligomers. The aggregation of monomeric alpha-synuclein to fibrils is deposited in Lewy bodies that have been reported to be expansive from neuron to neuron and from one region to another region of the brain. The aggregation of monomeric alpha-synuclein to oligomers induces the death of dopaminergic neurons that contain neuromelanin but are also capable of being released from one neuron to another neuron; therefore, alpha-synuclein cannot be the neurotoxin that induces single-neuron degeneration. DOPAL is generated in the process of dopamine degradation catalyzed by monoamine oxidase. In studies with postmortem material from patients with Parkinson’s disease, a decrease in the expression of the enzyme aldehyde dehydrogenase-1 was observed in comparison with control brains [66]. A decrease in the expression of aldehyde dehydrogenase implies an accumulation of DOPAL that can generate neurotoxic effects. However, the postmortem tissue is what survived the neurodegenerative process. Free neuromelanin is capable of inducing microglia activation and ultimately neurotoxicity [21]. However, the rupture of these double-membrane structures that contain neuromelanin would release large amounts of free neuromelanin that would have an expansive effect. Aminochrome is a transient neurotoxin that is formed in the synthesis of neuromelanin. In vitro studies using NMR showed that aminochrome is stable for 40 min before it begins to convert to 5,6-indolequinone, the precursor of neuromelanin [67]. Aminochrome forms adducts with proteins or can be reduced by flavoenzymes, which suggests that free aminochrome in one neuron only lasts for seconds, making it impossible for it to exhibit an expansive characteristic affecting neighboring neurons. Therefore, aminochrome may be the neurotoxin that induces the single-neuron neurodegeneration model since aminochrome induces alpha-synuclein aggregation, the protein degradation dysfunction of both the proteasomal and lysosomal systems, neuroinflammation, mitochondrial dysfunction, oxidative stress, and endoplasmic reticulum stress [23,68,69,70] (Figure 4).

## 6. Aminochrome

**Figure 5:**
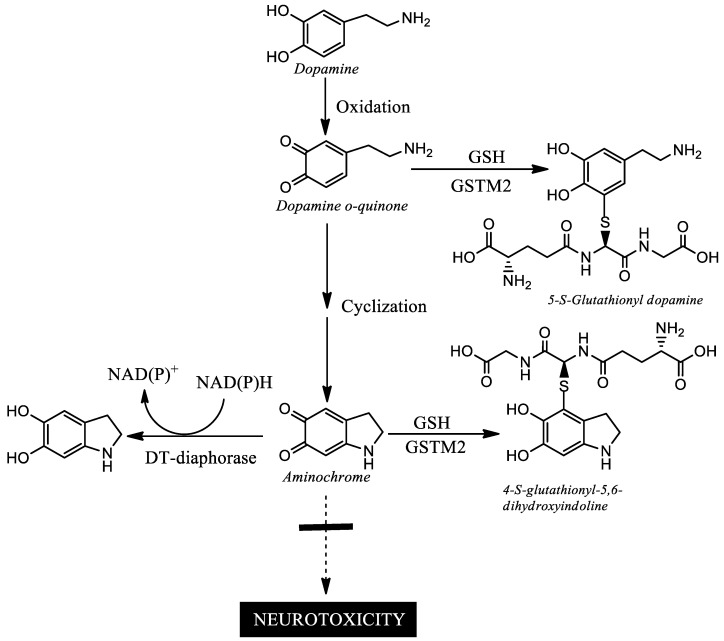
Neuroprotection from the neurotoxic effects of aminochrome. DT-diaphorase catalyzes the two-electron reduction of aminochrome to leukoaminochrome by preventing aminochrome from being reduced with one-electron by other flavoenzymes to leukoaminochrome ortho-semiquinone radical. Leukoaminochrome ortho-semiquinone radical is extremely reactive with oxygen generating a redox cycle that depletes NADH, dioxygen and induces oxidative stress. Glutathione transferase M2-2 catalyzes the conjugation of aminochrome to 4-S-glutathionyl-5,6-dihydroxyindoline that is resistant to biological agents (dioxygen, hydrogen peroxide and superoxide). In addition, glutathione transferase M2-2 catalyzes the conjugation of ortho-quinone dopamine to 5-S-glutathionyl dopamine which is degraded to 5-cysteinyl dopamine which has been detected in cerebrospinal fluid and neuromelanin (23).

Aminochrome-induced neurotoxicity. Aminochrome is the most stable and neurotoxic ortho-quinone formed during neuromelanin synthesis. Aminochrome is neurotoxic by inducing the formation of neurotoxic alpha-synuclein oligomers, the protein degradation dysfunction of both the proteasomal and lysosomal systems, mitochondrial dysfunction, oxidative stress, endoplasmic reticulum stress, and neuroinflammation [23,70]. In healthy elderly people, the formation of aminochrome during neuromelanin synthesis is not neurotoxic because there are two enzymes, DT-diaphorase and Glutathione transferase M2-2, that prevent aminochrome neurotoxic effects (50; Figure 5).

## 7. Conclusions

Iron plays a fundamental role in human life, but iron excess and deficiency can be related to different pathologies. A regulatory system exists to maintain iron homeostasis to prevent iron from inducing pathological effects. Iron is a transition metal that, in its reduced state (Fe^2+^), can induce the formation of reactive oxygen species, resulting in oxidative stress. Excess iron in transfusion-dependent thalassemia and pantothenate kinase-associated neurodegeneration has motivated the therapeutic use of iron chelators to avoid the neurotoxic effects of iron. For a long time, the existence of iron deposits in postmortem tissue of Parkinson’s patients has been interpreted as evidence of the neurotoxic role of iron in the neurodegenerative process of Parkinson’s disease. A clinical study with the iron chelator deferiprone in patients with Parkinson’s disease did not show positive effects, but rather, the clinical symptoms of the disease worsened. The negative result of this clinical study with patients with Parkinson’s disease with the chelator deferiprone and the lack of effect of exposure for decades in the Huasco population exposed to high iron concentrations suggest that iron does not play a fundamental role in the neurodegenerative process of the nigrostriatal system in Parkinson’s disease. However, the role that iron plays in the loss of dopaminergic neurons that contain neuromelanin in idiopathic Parkinson’s disease is controversial since there is a significant number of researchers who consider that iron plays a fundamental role in the degenerative process in patients with idiopathic Parkinson’s disease. The possibility that the failure of deferiprone in this clinical study depends on a disturbing action of this chelator on key proteins in intracellular iron homeostasis such as ferritin or ferroportin 1 is very difficult to think about, since deferiprone has demonstrated high efficacy in the treatment of pathologies with excess iron without affecting key proteins in iron homeostasis. The objective of this review is to introduce a different point of view to the discussion about the role of iron in idiopathic Parkinson’s disease. There is a long list of clinical studies based on exogenous neurotoxins such as MPTP and 6-hydroxydopamine that have failed [51,58,59,60,61]. This review introduces to the discussion of the role of iron in Parkinson’s disease the possible role of the single-neuromelanin-containing dopaminergic neuron degeneration model in explaining the failure of deferiprone in this clinical study with patients with Parkinson’s disease. This single-neuron degeneration model is completely opposite to the concept of preclinical models based on exogenous neurotoxins that have motivated these unsuccessful clinical studies. This may explain the extreme slowness of the degenerative process and progression of idiopathic Parkinson’s disease and the lack of effects of drugs tested in preclinical studies with exogenous neurotoxins that induce a rapid, expansive, and massive degenerative process.

## Data Availability

Not applicable.
